# 739 A Review of Current Methicillin-Resistant Staphylococcus Aureus Decolonization Practices in a Pediatric Burn Population

**DOI:** 10.1093/jbcr/irad045.214

**Published:** 2023-05-15

**Authors:** Tiffany Hockenberry, John Waterfield, Karen Richey, Kevin Foster

**Affiliations:** AZ Burn Center at Valleywise Health, Phoenix, Arizona; AZ Burn Center at Valleywise Health, Phoenix, Arizona; Arizona Burn Center Valleywise Health, Phoenix, Arizona; AZ Burn Center at Valleywise Health, Phoenix, Arizona; AZ Burn Center at Valleywise Health, Phoenix, Arizona; AZ Burn Center at Valleywise Health, Phoenix, Arizona; Arizona Burn Center Valleywise Health, Phoenix, Arizona; AZ Burn Center at Valleywise Health, Phoenix, Arizona; AZ Burn Center at Valleywise Health, Phoenix, Arizona; AZ Burn Center at Valleywise Health, Phoenix, Arizona; Arizona Burn Center Valleywise Health, Phoenix, Arizona; AZ Burn Center at Valleywise Health, Phoenix, Arizona; AZ Burn Center at Valleywise Health, Phoenix, Arizona; AZ Burn Center at Valleywise Health, Phoenix, Arizona; Arizona Burn Center Valleywise Health, Phoenix, Arizona; AZ Burn Center at Valleywise Health, Phoenix, Arizona

## Abstract

**Introduction:**

Methicillin-resistant staphylococcus aureus (MRSA) infections can result in severe sequela including death. Evidence-based practice guidelines exist for the universal decolonization of adult intensive care (ICU) patients. Evidence has not been established in the pediatric population, resulting in a knowledge gap regarding MRSA decolonization in children. Our practice is to test all patients upon admission for MRSA. Regardless of results, due to the immunocompromise of burn patients, all admissions are placed on a decolonization protocol that includes mupirocin ointment intranasally twice daily for five days. The repeated intranasal swabbing causes both discomfort and psychological distress for our pediatric patients. The purpose of this study was to evaluate current practice and the incidence of MRSA bacteremia in our pediatric population.

**Methods:**

This was a retrospective review of all pediatric patients admitted to our center between January 2018 and August 2022. Patients who had mupirocin ordered for decolonization (YO) were compared to those who did not (NO). Basic descriptive statistics were calculated.

**Results:**

A total of 508 individual patients had 519 admissions, YO 73% (n=379) NO 27% (n=140). There were no significant differences between groups for age, gender, race, ethnicity, admission diagnosis or mortality. Mupirocin was ordered at admission for 379 (73%) and administered per orders 74% of the time. YO patients were more likely to require ICU care 19% (n=73) vs 11% (n=16) (p=0.036) and mechanical ventilation 12% (n=46) vs 4% (n=6) (p=0.008). Length of stay (LOS) was longer for the YO group, 9.9 vs 5.11 days (p=0.008). The majority, 93% (n= 482) were admitted for burn injury, with a mean TBSA of YO 8.37 vs NO 5.86 (p=0.023). For YO, the mean number of doses to be administered, adjusted for LOS, was 6.71 vs actual doses given 3.8 (p < .0001). Overall incidence of MRSA positive blood cultures was 0.6% in the center but when controlled for setting it rose to 3% in ICU admissions. There were no positive blood cultures in non-ICU patients or the NO group. Three YOs had blood cultures positive for MRSA and had 80-90% of doses administered. Days to positive culture from admission were 6, 7 and 15.

**Conclusions:**

Our review demonstrated that mupirocin was frequently discontinued before a full course was administered, yet the overall incidence of MRSA bacteremia was low, with all cases occurring in the pediatric ICU setting. These findings indicate that our current protocol does not perform well in this population and needs refinement. Universal decolonization in non-ICU pediatric patients is likely unnecessary.

**Applicability of Research to Practice:**

An evidence-based protocol for children would improve antimicrobial stewardship in a vulnerable population and decrease psychological distress.

